# Disruption of CUL3-mediated ubiquitination causes proximal tubule injury and kidney fibrosis

**DOI:** 10.1038/s41598-019-40795-0

**Published:** 2019-03-14

**Authors:** Turgay Saritas, Catherina A. Cuevas, Mohammed Z. Ferdaus, Christoph Kuppe, Rafael Kramann, Marcus J. Moeller, Jürgen Floege, Jeffrey D. Singer, James A. McCormick

**Affiliations:** 1 Division of Nephrology and Hypertension, Oregon Health and Science University, Portland, Oregon USA; 2 Division of Nephrology and Clinical Immunology, University Hospital RWTH Aachen, Aachen, Germany; 3 Division of Pharmacology, Vascular & Metabolic Diseases, Erasmus MC, Rotterdam, Netherlands; 4 Department of Biology, Portland State University, Portland, Oregon USA

## Abstract

Cullin 3 (CUL3) is part of the ubiquitin proteasomal system and controls several cellular processes critical for normal organ function including the cell cycle, and Keap1/Nrf2 signaling. Kidney tubule-specific *Cul3* disruption causes tubulointerstitial fibrosis, but little is known about the mechanisms. Therefore, we tested the hypothesis that dysregulation of the cell cycle and Keap1/Nrf2 pathway play a role in initiating the kidney injury upon *Cul3* disruption. *Cul3* deletion increased expression of cyclin E and p21, associated with uncontrolled proliferation, DNA damage, and apoptosis, all of which preceded proximal tubule injury. The cdk2-cyclin E inhibitor roscovitine did not prevent the effects of *Cul3* deletion, but instead exacerbated the kidney injury. Injury occurred despite accumulation and activation of CUL3 substrate Keap1/Nrf2, proposed to be protective in kidney injury. *Cul3* disruption led to progressive interstitial inflammation, functionally relevant renal fibrosis and death. Finally, we observed reduced CUL3 expression in several AKI and CKD mouse models and in fibrotic human kidney tissue. These data establish CUL3 knockout mice as a novel genetic CKD model in which dysregulation of the cell cycle may play a primary role in initiating tubule injury, and that CUL3 dysregulation could contribute to acute and fibrotic kidney disease.

## Introduction

Sustained acute kidney injury (AKI) transitions into chronic kidney disease (CKD) with the development of tubulointerstitial fibrosis as the final endpoint^1^. However, the mechanisms involved are poorly understood, and their identification will be the first step towards an urgently needed therapy for the vast and growing CKD patient population.

Covalent linkage of ubiquitin to proteins (ubiquitination) plays a pivotal role in determining cellular function^2^, but its role in kidney fibrosis is largely unexplored^3^. Cullin 3 (CUL3), a member of the Cullin-RING ligase (CRL) family of ubiquitin ligases, is highly conserved and present in all human organs. Its disruption results in embryonic lethality^4,5^. CUL3 is absent from the glomerulus, but is expressed along all tubule segments, with highest mRNA and protein levels in proximal tubule^4,6^. In the CRL, CUL3 acts as a scaffold protein for the RING ubiquitin ligase, and for an array of substrate-binding adaptors that confer substrate-specificity.

CUL3 is involved in multiple intracellular pathways^7^ including those activated by Wnt/β-catenin^8^, Hedgehog/Gli^9^, NF-kB^10^, Notch^11^, Keap1/Nrf2^12^ and cell cycle proteins^5,13^, all reported to be critical in kidney injury and fibrosis. In humans, *Cul3* mutations are associated with renal cell carcinoma^14^ and cause the disease Familial Hyperkalemic Hypertension (FHHt, also known pseudohypoaldosteronism II)^15^. We previously generated doxycycline-inducible renal epithelia-specific *Cul3* knockout (KS-Cul3^−/−^) mice to investigate mechanisms underlying FHHt^6^. These mice display a complex phenotype, with increased activation of the thiazide-sensitive Na^+^-Cl^−^ cotransporter (NCC), and polyuria due to a loss of aquaporin-2 (AQP2). Chronically, they displayed histological signs of tubulointerstitial fibrosis and increased expression of the CUL3 substrate cyclin E, but the originating tubule segment, the pathways dysregulated, and the time-course of the development of renal injury were not determined. Therefore, the aims of this study were to i) identify the site of acute tubule injury upon *Cul3* deletion and to characterize the time-course of its transition into CKD, thus, establishing KS-Cul3^−/−^ mice as a novel genetic CKD model; ii) test the hypothesis that dysregulation of the cell cycle and Keap1/Nrf2 pathway precedes tubule injury, and that the cyclin E inhibitor roscovitine ameliorates kidney injury; iii) test the hypothesis that CUL3 plays a broader role in kidney disease by examining CUL3 expression in mouse models of AKI and CKD, and in fibrotic human samples.

## Results

### Increased proliferation and DNA damage precedes proximal tubule injury following *Cul3* disruption

**Figure 1:**
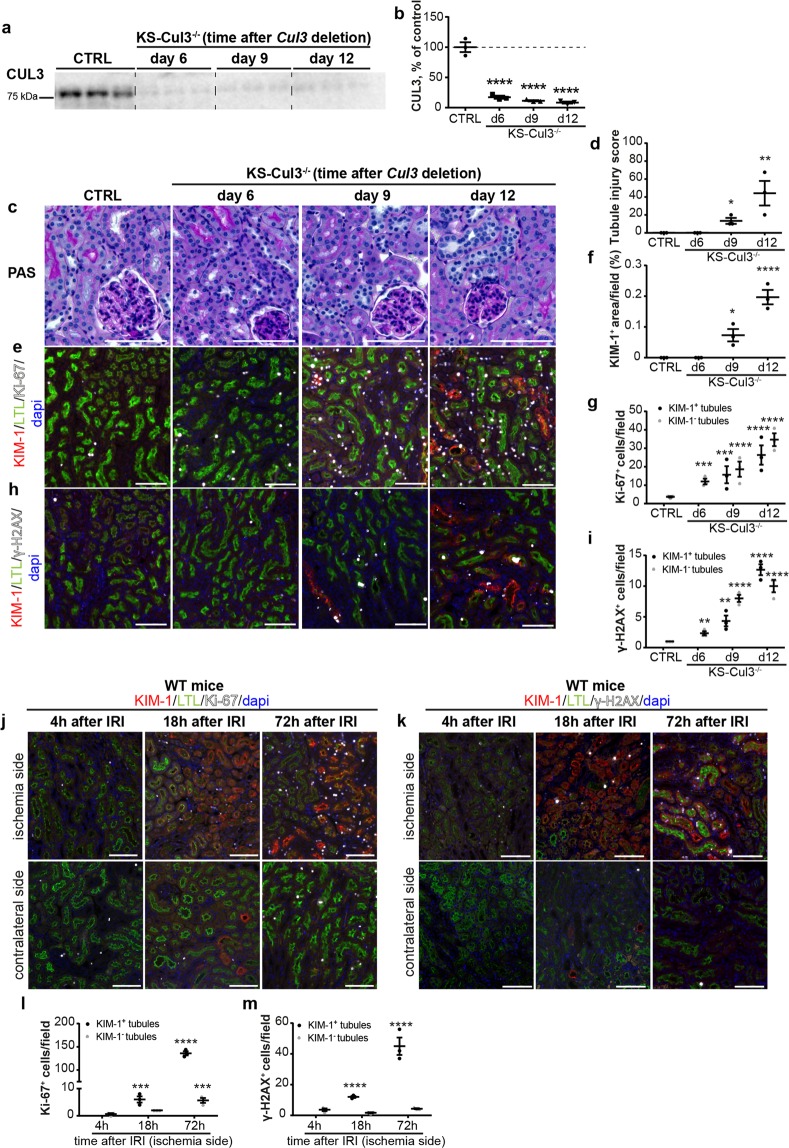
Comparison of tubule injury after kidney-specific *Cullin 3* deletion (KS-Cul3^−/−^) and ischemia/reperfusion injury (IRI). (**a**,**b**) Western blot of whole kidney lysate showed *Cul3* disruption after 6, 9 and 12 days of doxycycline administration. (**c**,**d**) Semi-quantitative analysis of periodic acid-Schiff staining revealed mild acute tubule injury upon *Cul3* deletion. (**e**,**f**,**h**) Immunofluorescence revealed expression of kidney injury molecule-1 (KIM-1) in lotus tetragonolobus lectin (LTL)^+^ proximal tubules (KIM-1^+^/LTL^+^) which was observed at days 9 and 12. From day 6 on, there were increased numbers of Ki-67^+^ proliferating cells in tubules (**e**,**g**), predominantly in proximal tubules, and interstitium, as well as DNA damage in proximal tubules (based on phosphorylated histone H2AX (γ-H2AX) staining (**h**,**i**), and this preceded expression of KIM-1. (**j**–**m**) In contrast to KS-Cul3^−/−^ mice, progressive proliferation (based on Ki-67 staining; **j**,**l**) or DNA damage (based on γ-H2AX staining; **k**,**m**) occurred concomitantly with KIM-1 expression in proximal tubules of WT mice after 18 h of ischemia/reperfusion injury (IRI). In the contralateral control kidney only a few proximal tubules were positive for KIM-1 and Ki-67 (**j**,**l**). Scale bars = 100 μm. Mean values are shown ± SEM. In (b, d, f) asterisks show significant differences between control and each KS-Cul3^−/−^ mice group (*n* = 3). In (g,i) statistical analysis were performed separately for KIM-1^+^ and KIM-1^−^ tubules and asterisks show significant differences between control (CTRL) and each KS-Cul3^−/−^ mice group (*n* = 3). In (l, m) asterisks show significant differences between group “4 h after IRI” and each later time points after IRI (*n* = 2–3). *P ≤ 0.05, **P ≤ 0.01, ***P ≤ 0.001, ****P ≤ 0.0001; Ordinary one-way ANOVA with Dunnett’s multiple comparisons test. Coomassie-stained gels used for Western blot normalization can be found in Supplementary Fig. S4. Western blots were cropped for clarity; uncropped images can be found in Supplementary Fig. S5.

To generate inducible renal-epithelia-specific *Cul3* knockout mice, *Cul3*^*flox/flox*^ mice were interbred *with Pax8-rtTA/LC1* transgenic mice as previously described^6,16^. In this system the reverse tetracycline transactivator (rtTA) is constitutively expressed under the control of the Pax8 promoter, which is active within the kidney along the entire renal epithelia. Doxycycline administered in drinking water binds to the rtTA which then promotes transcription of Cre recombinase from the LC1 transgene, leading excision of exons 4–7 at the “floxed” *Cul3* allele. Thus, disruption of *Cul3* is doxycycline-inducible, almost exclusively along the renal epithelia, in adult mice. To determine early effects of *Cul3* disruption and test the hypothesis that cell cycle dysregulation contributes to the initiation of injury, doxycycline was administered for 6, 9 or 12 days to disrupt *Cul3*. Maximal *Cul3* disruption was observed after 6 days (Fig. 1a,b). Mild acute tubule injury was detected after 9 and 12 days of Cul3 deletion, based on semi-quantification of periodic acid-Schiff (PAS)-stained slides (Fig. 1c,d). Haemotoxylin & Eosin (H&E) stained kidney sections are shown in Supplementary Fig. S1a. Immunofluorescence (IF) revealed kidney injury molecule-1 (KIM-1)^+^ signal in KS-Cul3^−/−^ mice at day 9 and 12, which coincided with lower Lotus tetragonolobus lectin (LTL)- signal in proximal tubules (PT), indicating acute tubule injury (Fig. 1e,f). From day 6 on, a progressive increase in Ki-67^+^ proliferating cells in KS-Cul3^−/−^ mice was observed (Fig. 1e,g). At day 6, the majority of Ki-67^+^ cells were localized in (LTL)-positive proximal tubules (PT) and Ki-67 was detected in both uninjured and injured PT, and in interstitial cells (Fig. 1e,g). Signal for the DNA damage marker γ-H2AX followed the same pattern as Ki-67 (Fig. 1h,i). IF for cleaved caspase-3 revealed that *Cul3* deletion induced apoptosis in PT cells at day 9 and 12 (see Supplementary Fig. S1b,c). *De novo* CD44 expression suggested that injury did not occur in glomeruli (no CD44^+^ parietal epithelial cells), but was restricted to PT (see Supplementary Fig. S1d).

A recent RNA-sequencing study reported that following ischemia/reperfusion injury (IRI), mRNA abundance of KIM-1 increased and peaked prior to induction of Ki-67^17^. This reported sequence appeared to be the opposite of changes in KIM-1 and Ki-67 protein in KS-CUL3^−/−^ mice. Therefore, we examined wild-type mice that had undergone unilateral IRI to permit direct comparison with our data from KS-Cul3^−/−^ mice at the protein level. 4 h after IRI, KIM-1 signal was still absent and only a low number of Ki-67^+^ cells was observed in both injured and contralateral non-injured kidney (Fig. 1j,l). 18 h after IRI, we observed an increase of Ki-67^+^ cells in KIM-1^+^ PT and adjacent interstitium in the injured kidney (Fig. 1j,l). Changes in γ-H2AX expression followed a similar pattern to that of Ki-67 following IRI (Fig. 1k,m). These observations were consistent with morphological signs of damage as seen by PAS and H&E staining (see Supplementary Fig. S2a,b). Thus, the injuries induced by *Cul3* disruption and IRI differ, with increased proliferation and DNA damage occurring *prior* to KIM-1^+^ PT injury in KS-CUL3^−/−^ mice, but occurring simultaneously following IRI. Uncontrolled proliferation, DNA damage and apoptosis may therefore drive the proximal tubule injury in KS-Cul3^−/−^ mice.

### Dysregulation of cell cycle proteins and Keap1-Nrf2 signaling precede injury following *Cul3* disruption

**Figure 2:**
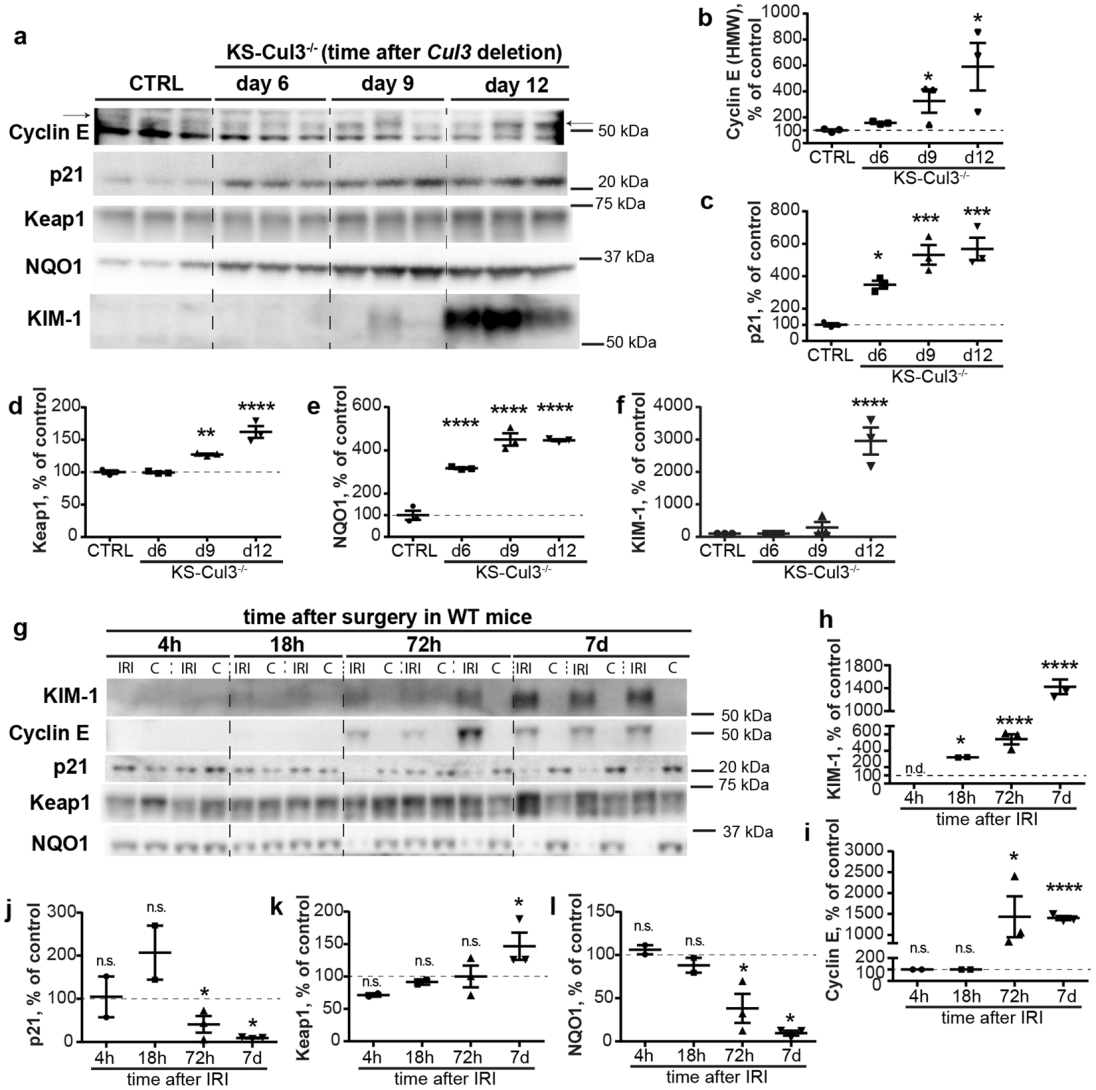
*Cul3* deletion and IRI have differential effects on cell cycle proteins and Keap-1/Nrf2 signaling. (**a**–**f**) Western blot analysis showed increased abundance of cyclin E and its inhibitor p21, as well as Keap1 and NQO1, a readout of Nrf2 signaling activation, after 6 or 9 days in kidney-specific *Cullin 3* knockout (KS-Cul3^−/−^) mice. KIM-1 signal was not detectable in control mice or 6 days after induction of *Cul3* deletion, and appeared at day 9 and 12 after *Cul3* disruption (**f**). (**g**–**i**) Compared to contralateral control kidney, Western blotting of wild-type kidney lysates revealed increased KIM-1 abundance 18 h after ischemia-reperfusion injury (IRI) (**g**,**h**), followed by increased abundance of cyclin E 72 h after IRI (**g**,**i**). (**g**–**l**) In contrast to KS-Cul3^−/−^ mice, KIM-1 induction was associated with decreased abundance of p21 (**g**,**j**), and increased Keap1 and decreased NQO1 (consistent with lower Nrf2 activity) after 72 h of IRI (**g**,**k**,**l**). Mean values are shown ± SEM. In (b-f, *n* = 3) asterisks show significant differences between control and each KS-Cul3^−/−^ mice group obtained from ordinary one-way ANOVA with Dunnett’s multiple comparisons test, with a single pooled variance. In (h-l, *n* = 2–3), protein abundance in injured kidney was compared to its contralateral control kidney using paired t-test for each time point. *P ≤ 0.05, **P ≤ 0.01, ***P ≤ 0.001, ****P ≤ 0.0001; Abbreviations: n.s., not significant; n.d., not detectable. Coomassie-stained gels used for Western blot normalization can be found in Supplementary Fig. S4. Western blots were cropped for clarity; uncropped images can be found in Supplementary Figs S5–S7.

CUL3 modulates abundances of the cell cycle protein cyclin E^5^, and of Keap1/Nrf2^18^, key players in oxidative stress response. These systems have been implicated in AKI and CKD^19,20^, and importantly, both cyclin E^5^ and Keap1^21^ are directly ubiquitinated and targeted for proteasomal degradation by CUL3. We hypothesized that both pathways are activated in KS-CUL3^−/−^ mice, but cyclin E effects on uncontrolled proliferation and DNA damage override protective effects of Keap1/Nrf2. Western blotting revealed significantly higher abundances of cyclin E and its inhibitor p21, as well as of Keap1 and NQO1, a readout of Nrf2 activity, within 12 days of Cul3 disruption (Fig. 2a–e). Consistent with IF (Fig. 1e,f,h), KIM-1 was only detected at significant levels at later time-points (Fig. 2a,f). Thus, while the Keap1-Nrf2 pathway was activated *prior* to injury in KS-CUL3^−/−^ mice, its potential protective effects were overridden by effects of *Cul3* disruption on other pathways.

Following IRI, consistent with IF (Fig. 1j,k), KIM-1 signal was detectable 18 h after IRI, and signal abundance increased further at days 3 and 7 (Fig. 2g,h). In contrast to KS-CUL3^−/−^ mice, changes in cyclin E, p21, Keap1, NQO1 occurred *after* KIM-1 induction (Fig. 2g–l). Furthermore, p21 and NQO1 abundances were significantly lower in the injured kidney than in the contralateral kidney (Fig. 2g,j,l).

### *Cul3* disruption causes sustained proximal tubule injury and inflammation

**Figure 3:**
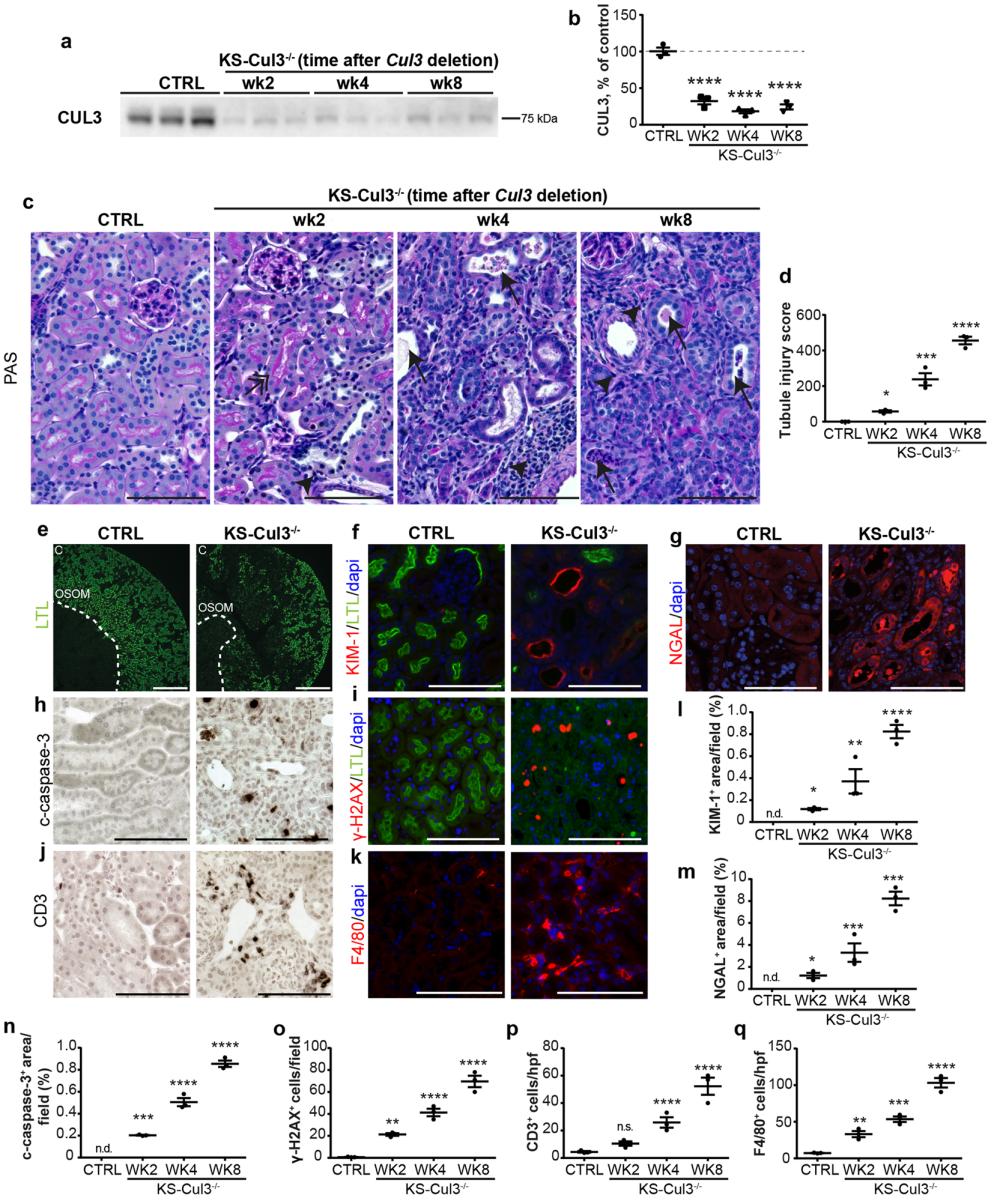
*Cul3* disruption causes sustained proximal tubule injury and inflammation. (**a**,**b**) To assess the progression of CUL3-mediated renal injury, *Cul3* disruption was induced by doxycycline administration, and kidney-specific *Cullin 3* knockout (KS-Cul3^−/−^) mice were evaluated after 2, 4 and 8 weeks. (**c**,**d**) Periodic acid-Schiff staining revealed progressive tubule injury characterized by cellular infiltration (arrowheads), very discrete subapical vacuolization (double-headed arrow) and dilated tubules with brush border loss, epithelial flattening and intratubular debris (arrows) upon *Cul3* disruption. (**e**) LTL- immunolabeling of kidney sections after 8 weeks of *Cul3* deletion indicated loss of brush border, i.e. a proximal tubule injury, in Cortex (C) and outer stripe of outer medulla (OSOM). (**f**–**q**) *Cul3* deletion caused progressive tubule injury and inflammation with positive staining for KIM-1 (**f**,**l**) and neutrophil gelatinase-associated lipocalin (NGAL) (**g**,**m**), cleaved capsase-3^+^ apoptosis (**h**,**n**), γ-H2AX^+^ DNA damage (**i**,**o**), CD3^+^ T-cell infiltration (j, p) and F4/80^+^ macrophage activation (**k**,**q**). Scale bars = 100 μm; except in (**e**) = 500 μm). *n* = 3. Mean values are shown ± SEM. Asterisks show significant differences between control and each KS-Cul3^−/−^ mice group. *P ≤ 0.05, **P ≤ 0.01, ***P ≤ 0.001, ****P ≤ 0.0001; ordinary one-way ANOVA with Dunnett’s multiple comparisons test. Abbreviations: n.s., not significant; n.d., not detectable. Coomassie-stained gels used for Western blot normalization can be found in Supplementary Fig. S4. Western blots were cropped for clarity; uncropped images can be found in Supplementary Fig. S7.

To determine the time-course of kidney injury, we analyzed mice 2, 4 and 8 weeks after initiating *Cul3* deletion (Fig. 3a,b). PAS staining showed progressive acute tubule injury characterized mainly by brush border loss, epithelial flattening, tubule dilation and intratubular debris (Fig. 3c,d). H&E staining showed similar kidney morphology (see Supplementary Fig. S3a). At week 8, there was a significant loss of LTA^+^ brush border, suggesting continued PT injury (Fig. 3e). This was supported by increased abundances of KIM-1, Neutrophil gelatinase-associated lipocalin (NGAL), cleaved caspase-3, γ-H2AX and Ki-67 over the time course (Fig. 3f–i,l–o; see Supplementary Fig. S3b–e). Infiltrating cells were positive for the T-cell marker CD3 (Fig. 3j,p) or macrophage marker F4/80 (Fig. 3k,q; see Supplementary Fig. S3f), but other infiltrating cell types may also have been present.

### *Cul3* disruption causes progression into renal fibrosis and persistent cell cycle dysregulation

**Figure 4:**
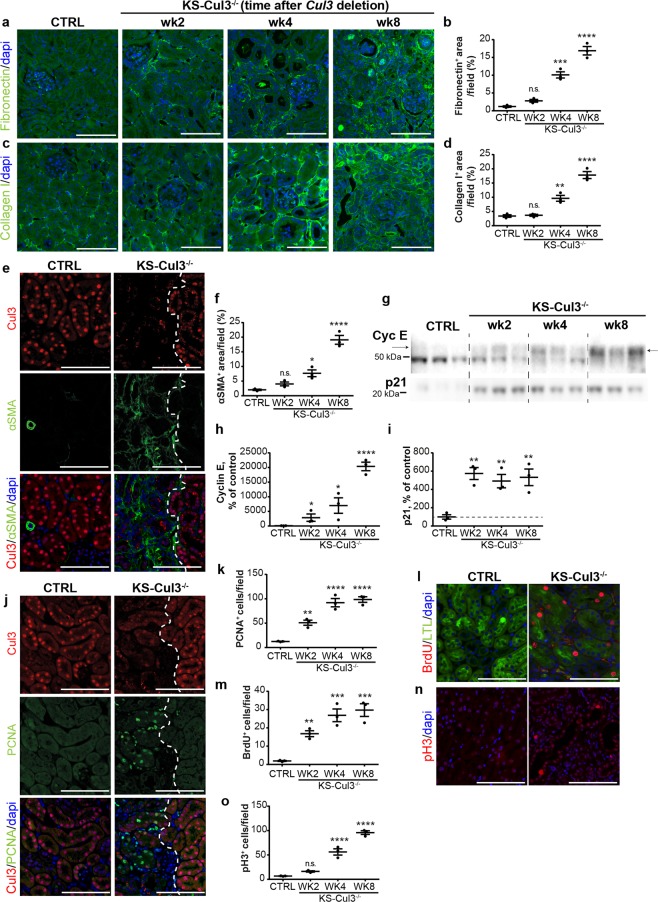
*Cul3* disruption causes progression into renal fibrosis and persistent cell cycle dysregulation. (**a**–**d**) Immunofluorescence staining revealed accumulation of fibronectin (**a**,**b**) and collagen I (**c**,**d**) in kidney-specific *Cullin 3* knockout (KS-Cul3^−/−^) mice. (**e**,**f**) *Cul3*-deficient tubules were adjacent to areas of increased alpha smooth muscle actin (α-SMA)^+^ extracellular matrix accumulation. (**g**–**i**) Western blotting showed persistent increase of cyclin E and p21 abundance in KS-Cul3^−/−^ mice up to at least 8 weeks. (**j**,**k**) Proliferating cell nuclear antigen (PCNA)^+^ cells, a marker for both DNA synthesis and DNA repair, were detectable within and nearby *Cul3*-deficient tubules. (**l**,**m**) Increased Bromodeoxyuridine (BrdU) incorporation indicated greater number of proliferating cells in S-phase upon *Cul3* deletion in LTL^+^ proximal tubules. (**n**,**o**) *Cul3* deletion was associated with increased number of tubule epithelial cells positive for phospho-Histone 3 (pH3), a marker for cells in G_2_ and M phase. Scale bars = 100 μm. Mean values are shown ± SEM. *n* = 3. Asterisks show significant differences between control and each KS-Cul3^−/−^ mice group. *P ≤ 0.05, **P ≤ 0.01, ***P ≤ 0.001, ****P ≤ 0.0001; ordinary one-way ANOVA with Dunnett’s multiple comparisons test. Abbreviation: n.s., not significant. Coomassie-stained gels used for Western blot normalization can be found in Supplementary Fig. S4. Western blots were cropped for clarity; uncropped images can be found in Supplementary Fig. S7.

IF staining revealed accumulation of fibronectin and collagen 1 in *Cul3* disrupted kidneys within 4 weeks (Fig. 4a–d). CUL3 protein was not completely ablated (Fig. 3a,b), and co-staining for CUL3 and alpha smooth muscle actin (α-SMA), another surrogate marker for fibrosis, revealed accumulation of α-SMA^+^ interstitial myofibroblasts in areas lacking CUL3 (Fig. 4e,f). These data indicate that disruption of *Cul3* causes sustained tubule injury with transition into CKD, thus establishing KS-Cul3^−/−^ mice as a genetically-inducible CKD model.

Cyclin E abundance increases upon IRI, peaks and then returns to pre-injury levels within 14 days^17^. In our model, *Cul3* disruption is irreversible leading to a persistent injury. Western blotting revealed that abundance of cyclin E continued to increase from weeks 2 to 8, while p21 abundance was maximal and did not increase further after 2 weeks (Fig. 4g,i). Compared to controls, IF for markers more specific to different cell cycle phases revealed upon *Cul3* disruption increased number of cells positive for PCNA (maximal at the S phase), Bromodeoxyuridine (BrdU) (S phase) and phospho-Histone 3 (pH3) (Fig. 4j–o), the latter of which has been reported as a marker for G2/M phase arrest^22^. These data suggest that *Cul3* disruption leads to persistent cell cycle dysregulation, causing tubule cells to enter into the cell cycle, and at least partially to arrest in the profibrotic G2/M phase^22^.

**Figure 5:**
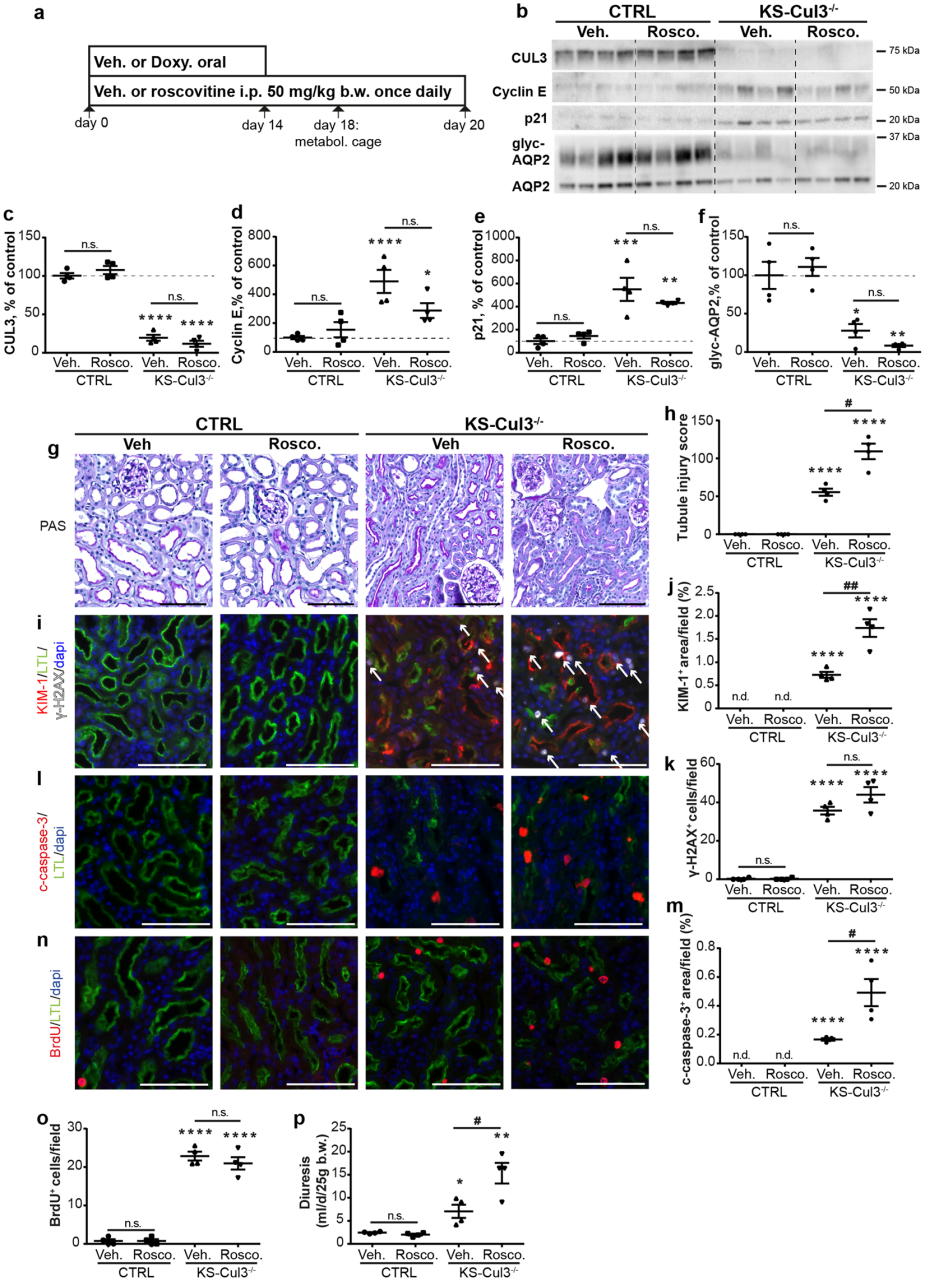
Cell cycle inhibitor roscovitine exacerbates kidney injury in KS-Cul3^−/−^ mice. (**a**) Roscovitine, an inhibitor of cyclin-dependent kinases including Cdk2-cyclin E, was co-administered with doxycycline during induction of *Cul3* deletion for 14 days, followed by roscovitine-injections only for 6 days. At day 18, metabolic cage experiments were performed to collect urine. Mice were sacrificed at day 20. (**b**–**f**) Western blotting revealed no effect of roscovitine (Rosco) on CUL3, cyclin E, p21 and AQP2 abundances in control and KS-Cul3^−/−^ mice. (**g**–**o**) In control mice, roscovitine did not cause tubule injury (based on semi-quantification of PAS-stained slides and KIM-1 expression; **g**–**j**) or DNA damage (based on γ-H2AX signal (white arrows); **i**,**k**) or apoptosis (based on cleaved caspase-3 signal; **l**,**m**), and had no effect on proliferation rate (based on BrdU signal; **n**,**o**). However, in the absence of CUL3, roscovitine further increased the kidney injury (**g**–**j**) and apoptotic response (**l**,**m**). (**p**) Roscovitine exacerbated polyuria in KS-Cul3^−/−^ mice. Scale bars = 100 μm. Mean values are shown ± SEM. One-way ANOVA with Dunnett’s multiple comparisons test were used; *represents statistical significant interaction between control group (vehicle-treated control mice) and each other group, ^#^highlights statistical differences between vehicle and roscovitine-treated mice. n = 4. *P ≤ 0.05, **P ≤ 0.01, ***P ≤ 0.001, ****P ≤ 0.0001; # ≤ 0.05, ## P ≤ 0.01. Abbreviations: n.s., not significant; Doxy, doxycycline, metabol. cage, metabolic cage. Coomassie-stained gels used for Western blot normalization can be found in Supplementary Fig. S4. Western blots were cropped for clarity; uncropped images can be found in Supplementary Fig. S8.

Since cyclin E abundance was increased in KS-CUL3^−/−^ mice, we determined whether roscovitine, an inhibitor of cyclin-dependent kinases (cdk) including Cdk2-cyclin E, could ameliorate the effects of *Cul3* disruption by co-administration with doxycycline during induction of *Cul3* deletion (Fig. 5a). Roscovitine had no effect on abundances of CUL3, cyclin E or p21 in control or KS-CUL3^−/−^ mice compared with vehicle-treated mice (Fig. 5b–e). *Cul3* disruption leads to polyuria due to loss of AQP2^6^. Roscovitine-treated KS-Cul3^−/−^ mice tended to have lower AQP2 levels than vehicle-treated KS-Cul3^−/−^ mice (Fig. 5b,f), Roscovitine exacerbated the effects of *Cul3* disruption on kidney injury (Fig. 5g–j), and apoptosis (Fig. 5l,m). Roscovitine did not significantly affect DNA damage (Fig. 5i,k) and proliferation in KS-CUL3^−/−^ mice (Fig. 5n,o). Roscovitine exacerbated the effects of *Cul3* disruption on polyuria (Fig. 5p).

### Chronic *Cul3* disruption results in death from renal failure

**Figure 6:**
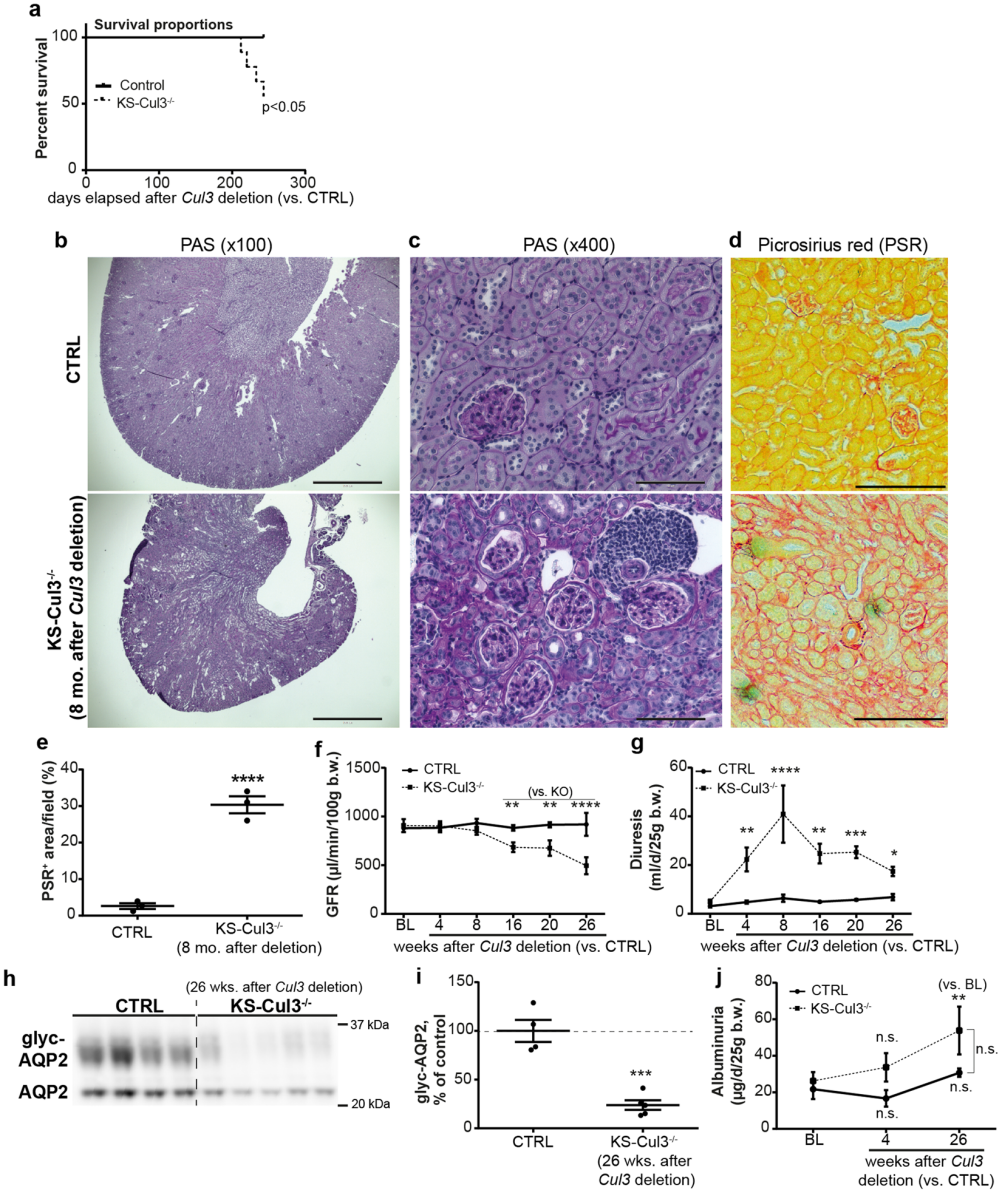
*Cul3* disruption caused death from progressive kidney fibrosis. (**a**) Kidney-specific *Cullin 3* knockout (KS-Cul3^−/−^) mice (3 females, one male) spontaneously died 7–8 months after *Cul3* disruption (8 CTRL vs. 9 KS-Cul3^−/−^ mice; log rank test; experiment was stopped after 4 deaths and samples were obtained from remaining mice for further analysis). (**b**,**c**) Periodic acid-Schiff staining of kidney tissue from remainder of (**a**) revealed kidney atrophy, cell infiltration, tubule dilation, epithelial simplification and interstitial expansion. Scale bars = 1000 μm (**b**) and 100 μm (**c**–**e**) Picrosirius red staining (PSR) revealed increased collagen deposition. Scale bars = 200 μm. (**f**) Measurement of glomerular filtration rate (GFR) with fluorescence-labeled sinistrin-administration and its transcutaneous detection showed progressive decline of kidney function after 16 weeks of *Cul3* deletion. (**g**–**i**) *Cul3* deletion caused decreased abundance of AQP2 and polyuria peaked at 8 weeks, but then despite low AQP2 levels diuresis declined, likely due to renal failure. (**j**) Albumin excretion was not significantly different between control and KS-Cul3^−/−^ mice. Mean values are shown ± SEM. Asterisks show significant differences between control and KS-Cul3^−/−^ mice at given time point obtained by unpaired t-test (*n* = 4–5). *P ≤ 0.05, **P ≤ 0.01, ***P ≤ 0.001, ****P ≤ 0.0001; Abbreviations: n.s., not significant; glyc, glycosylated; BL, baseline; b.w., bodyweight. Coomassie-stained gels used for Western blot normalization can be found in Supplementary Fig. S4. Western blots were cropped for clarity; uncropped images can be found in Supplementary Fig. S9.

7–8 months after induction of *Cul3* disruption, mice displayed increased mortality (Fig. 6a). We sacrificed the remainder of the cohort for analyses. Blood chemistry revealed hyperkalemia, anemia, and elevated blood urea nitrogen (Table 1), typical features of late CKD. Histology revealed severe global renal fibrosis and mild to moderate glomerulosclerosis (Fig. 6b,c), and Picrosirius red staining revealed high levels of collagen deposition (Fig. 6d,e). Glomerular filtration rate (GFR) measurements revealed a progressive decline in kidney function 16 weeks after induction of *Cul3* disruption (Fig. 6f). Diuresis peaked 8 weeks after *Cul3* disruption then decreased despite persistently lower AQP2 abundance (Fig. 6g–i). Thus, the declining diuresis is likely due to renal failure. Urinary albumin did not differ at baseline or at 4 or 26 weeks between controls and KS-CUL3^−/−^ mice, but in KS-CUL3^−/−^ mice it was significantly elevated from baseline at 26 weeks (Fig. 6j). This suggests that glomerular damage is not a primary feature of the phenotype, consistent with *Cul3* disruption specifically along renal epithelia.

**Table 1:** Measured blood parameters 8 months after *Cul3* disruption.

Blood Parameters	CTRL	KS-Cul3^−/−^	P value
Sodium (mmol/L)	146 ± 0.2	153 ± 1.8	**0**.**001**
Potassium (mmol/L)	4.2 ± 0.1	5.9 ± 0.5	**0**.**003**
Chloride (mmol/L)	111 ± 0.9	109 ± 1.1	0.147
Ionized Calcium (mmol/L)	1.3 ± 0	1.1 ± 0	**0**.**006**
Total Carbon Dioxide (mmol/L)	23 ± 1.5	28 ± 0.6	**0**.**027**
Blood Urea Nitrogen (mg/dL)	22 ± 1.5	129 ± 11.2	**0**.**001**
Creatinine (mg/dL)	0.2 ± 0	0.8 ± 0.1	**0**.**001**
Hematocrit (% PCV)	35 ± 1.7	22 ± 2.7	**0**.**001**
Hemaglobin (g/dL)	11.9 ± 0.6	7.4 ± 0.9	**0**.**001**
Anion Gap (mmol/L)	17 ± 1.1	21 ± 0.9	0.218
Body weight (g)	30.1 ± 1.9	16.8 ± 1.2	**0**.**001**

KS-Cul3^−/−^ mice display CKD phenotype. Hyperkalemia, renal failure, anemia and other abnormal blood values 8 months after *Cul3* deletion. Data represent the mean ± SEM. P values were calculated by unpaired, two-tailed, t test obtained from 8 CTRL vs. 5 KS-Cul3^−/−^ mice.

### Decreased nuclear CUL3 localization is associated with renal injury in animal models and humans

**Figure 7:**
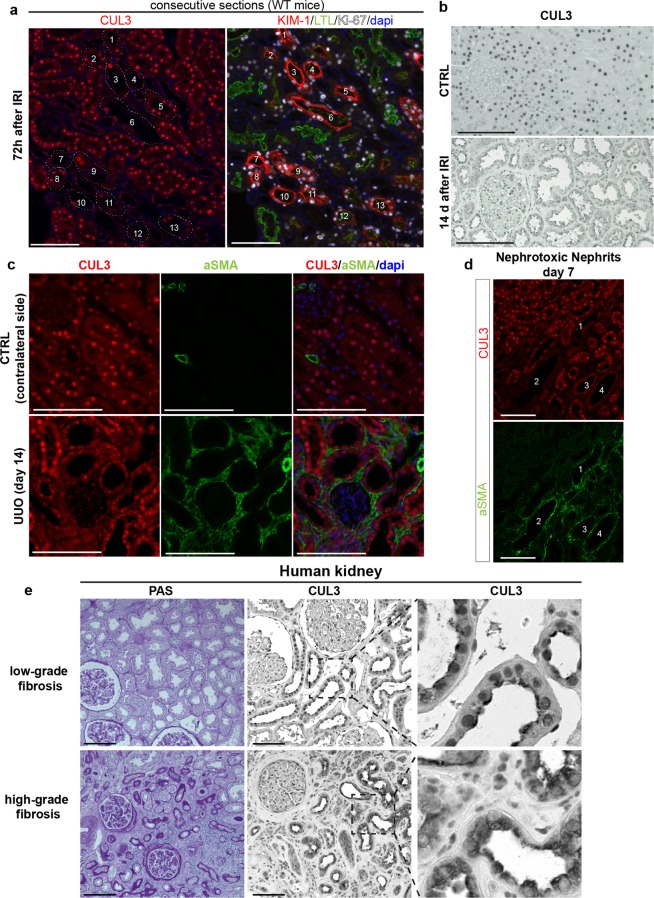
CUL3 expression is dysregulated in AKI/CKD mouse models and human kidney tissue. (**a**) Immunofluorescence of consecutive kidney sections from control mice 72 h after IRI revealed decreased signal for CUL3 in most KIM-1^+^ tubules. *n* = 3. (**b**) Immunohistochemistry revealed strong CUL3 signal in sham mice in contrast to tubule cells 14 days after IRI. *n* = 3. (**c**) 14 days after unilateral ureteral obstruction (UUO), tubules had lower CUL3 signal and were adjacent to areas of increased alpha smooth muscle actin (α-SMA)^+^ extracellular matrix accumulation. *n* = 3. (**d**) Similar to (**c**), 7 days after injection of nephrotoxic serum, tubules had lower CUL3 signal and were adjacent to α-SMA^+^ areas. *n* = 3. (**e**) Human kidney tissue was obtained from tumour nephrectomy specimens and Periodic acid-Schiff stained sections of non-tumour renal tissue were assigned to the low-grade versus high-grade fibrosis groups. Immunohistochemistry revealed reduced CUL3 signal in high-grade fibrosis compared to low-grade fibrosis group. *n* = 3. Scale bars = 100 μm.

Since lower CUL3 signal correlated with tubule injury and fibrosis, we next asked whether CUL3 expression might be affected in commonly used AKI/CKD mouse models, and in human kidney fibrosis. IF on consecutive sections 72 h after IRI revealed decreased signal for CUL3 in most KIM-1^+^ tubules in the injured kidney (Fig. 7a). However, Ki-67 expression did not completely correlate with lower CUL3 signal (Fig. 7a). Decreased CUL3 signal was also observed 14 days after IRI (Fig. 7b). Furthermore, following unilateral ureteral obstruction (UUO) or nephrotoxic nephritis (NTN), tubules with less CUL3 signal were surrounded by α-SMA^+^ areas (Fig. 7c,d). In human kidney, lower nuclear CUL3 signal was observed in areas with high-grade fibrosis than in areas with low-grade fibrosis (Fig. 7e).

## Discussion

We found that induced tubule-specific CUL3 deletion in adult mice caused sustained proximal tubule injury, followed by interstitial inflammation, progressive fibrotic renal disease and death. This establishes KS-CUL3^−/−^ mice as a novel animal model of CKD and implicates CUL3 as major upstream player in several pathways within the kidney. *Cul3* disruption caused dysregulation of the cell cycle characterized by uncontrolled proliferation, DNA damage and apoptosis *prior* to tubule injury, and increased Nrf2 activity upon *Cul3* deletion could not prevent the development of fibrosis. In contrast, ischemia-induced tubule injury preceded and triggered changes in cell cycle proteins and Keap1-Nrf2 signaling, suggesting the mechanisms involved in tubule injury differ between the two models.

Since CUL3-mediated degradation of cyclin E is required for cells to maintain a quiescent state^5^, loss of CUL3 in renal epithelia resulted in constant overexpression of cyclin E and hence proliferation. Deregulation of cyclin E turnover results in chromosomal instability, DNA damage and apoptosis^23,24^, which were all observed following *Cul3* disruption. These findings are consistent with previous reports in which fibroblast or hepatocyte-specific deletion of *Cul3* resulted in proliferation and polyploidy as well as chromosome breakage, micronuclei formation, and apoptosis^24,25^. DNA damage can itself induce transcription of p21, causing cell cycle arrest by inhibition of cyclin E and other targets^26^. We observed increased abundance of p21 upon *Cul3* deletion. Most evidence to-date suggests that p21 is a substrate of Cul1^skp2^ and Cul4A^Cdt2^-mediated degradation^27,28^, so it remains to be determined whether this was a direct result of *Cul3* deletion or secondary to DNA damage and other cell-cycle effects. Interestingly, it has been shown that increased expression of p21 in proximal tubules results in increased TGF-β mRNA, and vice versa, mediating kidney fibrosis^29^. IRI induced a proliferative response, supporting the well-characterized role of the cell cycle in adaptive (and maladaptive) repair after kidney injury^20^. In contrast, persistent proliferation was induced *prior* to kidney injury in KS-CUL3^−/−^ mice, which likely causes cell cycle dysregulation and DNA damage, and may drive the injury. However, roscovitine did not have beneficial effects by suppressing cell cycle entry in KS-CUL3^−/−^ mice, but exacerbated kidney injury and apoptosis. Roscovitine is a broad-range purine analog inhibitor, which in addition to inhibiting cdk2/cycline E, targets other cell cycle components including cdk1, cdk5, cdk7, and cdk9^30^. In cancer cell lines, which are highly proliferative, roscovitine not only arrests the cell cycle, but also induces apoptosis^31^. Therefore, since *Cul3* disrupted tubules displayed uncontrolled proliferation, roscovitine may have caused further cell cycle dysregulation and apoptosis in these mice. This speculation is supported by the observation that roscovitine had no effect in control mice, consistent with previous reports in which administration of roscovitine was well-tolerated in healthy mice^31,32^.

CUL3 and its adaptor Keap1 mediate degradation of Nrf2, a master regulator of anti-oxidant responses^18^. Mouse models and clinical trials in humans suggest that activation of Nrf2 or inhibition of Keap1 ameliorate AKI severity and slow CKD progression^33–35^. However, the recent demonstration that Nrf2 activation can result in chronic inflammation and kidney fibrosis by NFκB^10^ and the NLRP3 inflammasome^36^ questions the consensus that Nrf2 exerts beneficial effects in renal fibrosis. We show that Keap1/Nrf2 activity is elevated in KS-CUL3^−/−^ mice, suggesting a central role for CUL3 in regulating anti-oxidant responses in the kidney. Furthermore, other pathways (e.g. Hedgehog/Gli) related to kidney fibrosis are regulated by CUL3, and further studies including transcriptomic and proteomic analyses will be required to assess the full effects of *Cul3* disruption, particularly in the early phase. A recent transcriptomics analysis reported that the chemokine CXCL12 induced CUL3 expression in a human fibroblast cell line, and this was associated with procollagen secretion^37^. Pharmacological blockade of CUL3 activation prevented CKCL12-induced procollagen secretion, suggesting a direct role for CUL3^37^. However, in this study^37^, CUL3 was upregulated in fibroblasts while here we disrupted *Cul3* in tubule epithelia, so it remains to be determined whether selective inhibition of CUL3 in e.g. (myo-)fibroblasts may ameliorate kidney fibrosis upon injury.

Similarly to CUL3 knockout mice, in other mouse models of acute or chronic injury, reduced CUL3 signal was associated with increased proximal tubule injury and fibrosis, and reduced CUL3 was also observed in highly fibrotic human tissue. Altered CUL3 activity may therefore contribute to the overall proliferative response and activation of several profibrotic pathways in injury. Recent work highlights the proximal tubule as the primary sensor and effector of the AKI-CKD transition^38^. Our data support the idea that an isolated tubule injury can cause peritubular inflammation, interstitial fibrosis, and renal functional decline. Various mouse models have identified several pathways that may be targeted to slow down progression of CKD, but good animal models of tubulointerstitial fibrosis are lacking^39^. Current genetic models are mainly restricted to disruption of glomerular proteins, and the few tubule-specific CKD models involve non-inducible conditional genetic modifications or modifications in non-proximal tubule segments^39–42^. Here, we present a mouse model which permits the induction of sustained proximal tubule injury within 1–2 weeks in adult mice. One limitation is that *Cul3* deletion along the entire nephron leads to a complex phenotype including polyuria, so a future refinement will be to use an inducible proximal tubule-specific Cre driver^43^. Despite this limitation, our model recapitulates many features of CKD seen in humans and may provide insights into how tubular injury facilitates progressive kidney fibrosis. Shifting our attention from the late features of CKD to the early responses of the proximal tubule could lead to novel therapeutic targets, and identification of the CUL3 substrates involved may serve as a starting point.

## Methods

### Animals

Animal studies were performed in adherence to the National Institutes of Health Guide for the Care and Use of Laboratory Animals and approved by the OHSU Institutional Animal Care and Use Committee (protocol IP00286) and by LANUV (Germany) (9.93.2.10.35.07.041, 8.87–50.10.35.08.254). All experiments and methods were performed in accordance with relevant guidelines and regulations. Inducible renal epithelia-specific *Cul3* knockout mice were generated by interbreeding Pax8-rtTA/LC1 transgenic mice with *Cul3*^*flox/flox*^ mice as reported^6^. *Cul3* disruption was induced by administration of 2 mg/ml doxycycline in drinking water with 5% sucrose (vehicle) up to 2 weeks. Controls received vehicle for the same duration as the doxycycline exposure. Males and females were both used, were 8 to 14 weeks of age at time of doxycycline, vehicle or roscovitine treatment, and had ad libitum access to drinking water and standard chow.

### Murine renal injury models

Wild type C57BL/6 mice were used between 8 to 14 weeks of age for injury studies. For IRI, the left kidney was exposed through flank incision and was subjected to ischemia by clamping the left renal pedicle for 28 minutes at 36.5 °C to 37.5 °C body temperature^44^. The clamp was removed and the kidney was reperfused. Mice were sacrificed 4 h, 18 h, 3 days, 7 days, and 14 days post-surgery. For UUO, left kidney was exposed through flank incision and the ureter was tied off at the level of the lower pole with two 4.0 silk ties^45^. Mice were sacrificed 14 days post-surgery. Nephrotoxic nephritis was induced by a single intraperitoneal injection of 5 mg/g nephrotoxic serum^46^. Mice were sacrificed 7 days post-injection.

### Human samples

Use of human kidney samples was approved by the ethics committee of the University Hospital RWTH Aachen (EK 221/16) and informed consent was obtained from all participants. All experiments and methods were performed in accordance with relevant guidelines and regulations. Research was conducted in accordance with the Declaration of Helsinki. Unaffected renal tissue from patients with renal carcinoma was provided by the Eschweiler-Aachen Biobank. As described^44^, fibrosis severity was scored in PAS-stained sections and samples were stratified into low-grade fibrosis (fibrosis grade ≤20%) and high-grade fibrosis (fibrosis grade ≥40%) groups. Blinded to clinical data, 3 samples per group were selected, and sections stained for CUL3.

### Antibodies and chemicals

A detailed list of antibodies and chemicals is provided in Supplementary Table S1.

### Roscovitine

Roscovitine was dissolved in 5% DMSO, 50% PEG300, 45% H_2_O and injected i.p. at 50 mg/kg body weight once daily for 20 days. The dose was chosen based on previous reports^47,48^.

### Western Blot

As described^49^, 40 μg protein of whole kidney lysates were separated on 4–12% Bis-tris acetate gels and transferred to polyvinylidene fluoride membrane. Membranes were blocked with 5% non-fat milk in TBS-Tween, followed by incubation with primary antibody and HRP-coupled secondary antibody. Using Western Lightning ECL, signal was detected with a Syngene Pxi4 imager, and densitometry was performed with ImageJ. Actin abundance can change in response to experimental manipulation, so we normalized by densitometric quantitation of total protein via Coomassie staining^49,50^. Coomassie gels are shown in Supplementary Fig. S4.

### Histology, Immunofluorescence (IF) and Immunohistochemistry

Kidneys were perfusion-fixed with 4% paraformaldehyde, and 5 µm paraffin sections were stained with H&E, PAS, and Picrosirius Red by the OHSU Histopathology Core. Acute tubule damage was semi-quantified on PAS-stained slides by scoring extent of (a) brush border loss in proximal tubules, (b) epithelial cell flattening and (c) vacuolization^51^. Each phenomenon was separately scored as: 0, absent; 1, slightly present; 2 moderately present; and 3, severely present. The percentage representation of each score was estimated and multiplied by the score itself (i.e. leading to values in the range 0–300; e.g. 100% severely present brush border loss would reach the score 3 × 100 = 300) The tubule injury score of each animal was expressed by adding the scores for brush border loss, epithelial flattening and vacuolization (i.e. leading to values in the range 0–900). For IF, sections were incubated for 2 h at room temperature with primary antibodies in 1%BSA/PBS, followed by Cy2, Cy3 or Cy5-conjugated secondary antibodies (all 1:500, ThermoFisher Scientific) for 1 h at room temperature, and stained with DAPI in mounting medium. Immunohistochemistry was performed with Vectastain ABC Kit, according to the manufacturer’s instructions.

### Blood analysis

Blood was collected via cardiac puncture and loaded into a Chem8+cartridge for electrolyte measurement by i-STAT analyzer (Abbot Point of Care Inc.).

### Glomerular filtration rate (GFR) and urinalysis

GFR was measured by determining elimination of fluorescein isothiocyanate (FITC)-sinistrin transcutaneously as described^52^. A fluorescence detector (NIC-Kidney; Mannheim Pharma & Diagnostics GmbH, Germany) was placed on a depilated region of the mouse’s back and FITC-sinistrin (75 mg/kg body weight) was injected retro-orbitally. Data were acquired in conscious freely-moving mice for 60–120 min and GFR calculated using the half-life (t_1/2_). 36 h after GFR measurements, 24 h urine was collected in metabolic cages (Hatteras Inc.) to measure volume, and albumin excretion (Albuwell M kit, Exocell) with modifications of urine dilution to account for increased diuresis in KS-Cul3^−/−^ mice.

### Imaging

Fluorescence images were captured with ZEISS AXIO Imager M2 or Keyence BZ-9000 microscopes. Light microscopy images were acquired using an EVOS FL auto-imaging system (Thermo Fischer Scientific). 12 images (200x field or 400x high-power-field, hpf) were randomly taken per kidney cortex and outer medulla. Area fractions and number of cells were calculated using ImageJ and expressed as (%) or number of cells per field.

### Statistical analyses

All values are expressed as means ± SEM. For comparison of two groups, two-tailed, unpaired, t-test was used. Protein abundance in IRI-injured kidney was compared to its contralateral control kidney using paired t-test for each time point. Comparison of several groups was performed using ANOVA with Dunnett’s multiple comparisons test. All tests were two-tailed and P < 0.05 was considered significant. All analyses were performed using GraphPad Prism version 6.01.

## Supplementary information

Supplementary Information

## Data Availability

All data generated or analysed during this study are included in this published article (and its Supplementary Information Files).
