# Supplementary material for: Disruption of CUL3-mediated ubiquitination causes proximal tubule injury and kidney fibrosis
Source: Sci Rep. 2019 Mar 14;9:4596. doi: 10.1038/s41598-019-40795-0 (PMC6418206; doi:10.1038/s41598-019-40795-0)
Supplement: Supplementary file 1 — Supplementary Information [file 41598_2019_40795_MOESM1_ESM.pdf]

Disruption of CUL3-mediated ubiquitination causes proximal tubule injury and kidney fibrosis

Dr. Turgay Saritas, Dr. Catherina A. Cuevas, Dr. Mohammed Z. Ferdaus, Dr. Christoph Kuppe, Prof. Rafael Kramann, Prof. Marcus J. Moeller, Prof. Jürgen Floege[2], Dr. Jeffrey D. Singer, Dr. James A. McCormick.

Supplementary Figure S1. Related to Figure 1. *Cul3* disruption causes proximal tubule injury and apoptosis.

Supplementary Figure S2. Related to Figure 1. Histology of wild-type mice kidneys after ischemia/reperfusion injury (IRI).

Supplementary Figure S3. Related to Figure 3 and 4. Tubule injury, proliferation and apoptosis upon *Cul3* deletion.

Supplementary Figure S4. Coomassie-stained gels related to immunoblots shown in main manuscript.

Supplementary Figure S5. Uncropped Western blot gels shown in main manuscript Fig. 1 and Fig. 2.

Supplementary Figure S6. Uncropped Western blot gels shown in main manuscript Fig. 2.

Supplementary Figure S7. Uncropped Western blot gels shown in main manuscript Fig. 2, Fig. 3 and Fig 4.

Supplementary Figure S8. Uncropped Western blot gels shown in main manuscript Fig. 4 and Fig. 5.

Supplementary Figure S9. Uncropped Western blot gel shown in main manuscript Fig. 6.

Supplementary Table S1. List of reagents and resources used in this study.

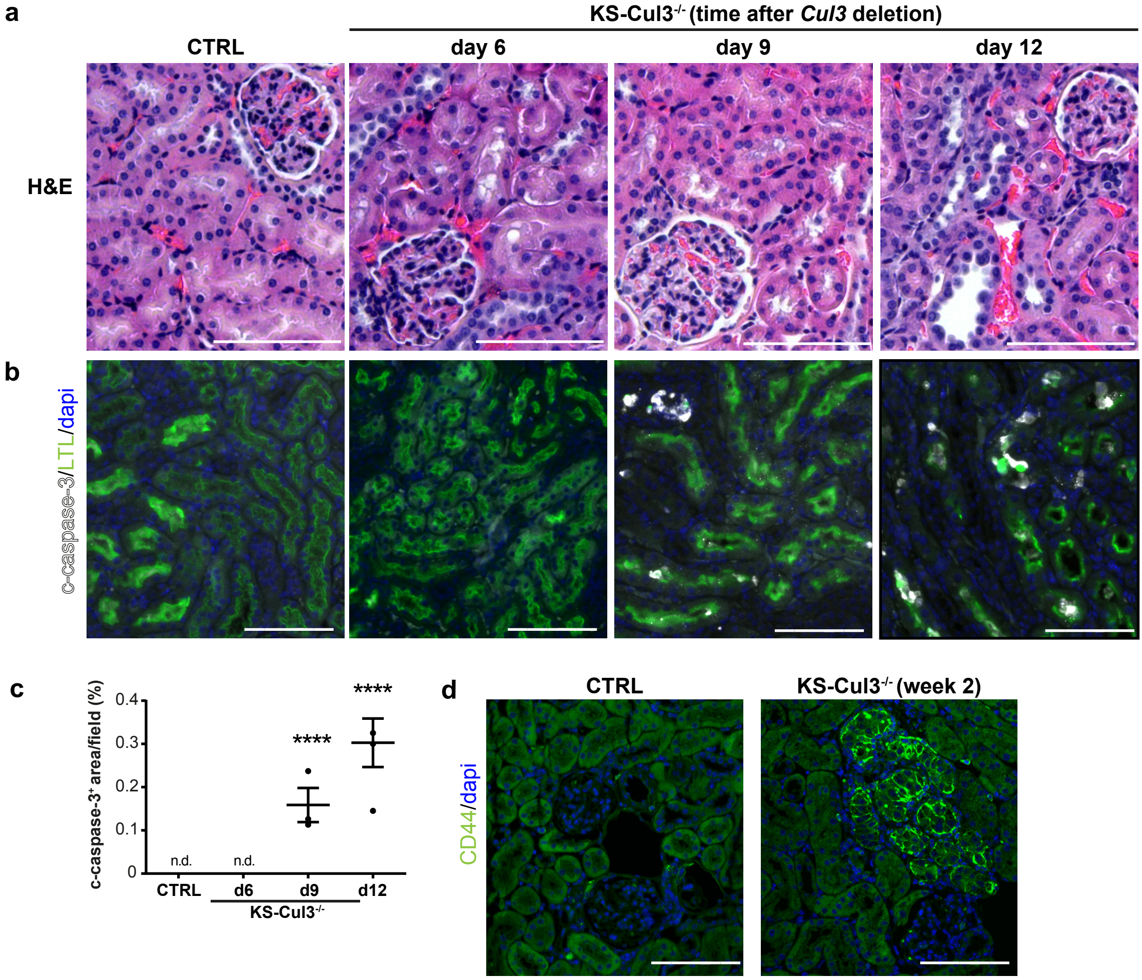

Supplementary Figure S1. Related to Figure 1. *Cul3* disruption causes proximal tubule injury and apoptosis.

(a) Haemotoxylin and Eosin (H&E) staining revealed no obvious morphological changes after 6, 9 and 12 upon *Cul3* deletion. **(b, c)** Immunofluorescence showed cleaved-caspase-3$^+$ apoptotic cells in LTL$^+$ proximal tubules after 9 and 12 days of *Cul3* deletion. **(d)** CD44 expression, which is generally absent, is markedly enhanced, particularly in injured proximal tubular epithelial cells upon 2 weeks of *Cul3* deletion. n = 3. Scale bars = 100 µm. Mean values are shown ± SEM. In (c) asterisks show significant differences between control and each KS-Cul3$^{-/-}$ mice group. ****P ≤ 0.0001; Ordinary one-way ANOVA with Dunnett's multiple comparisons test.

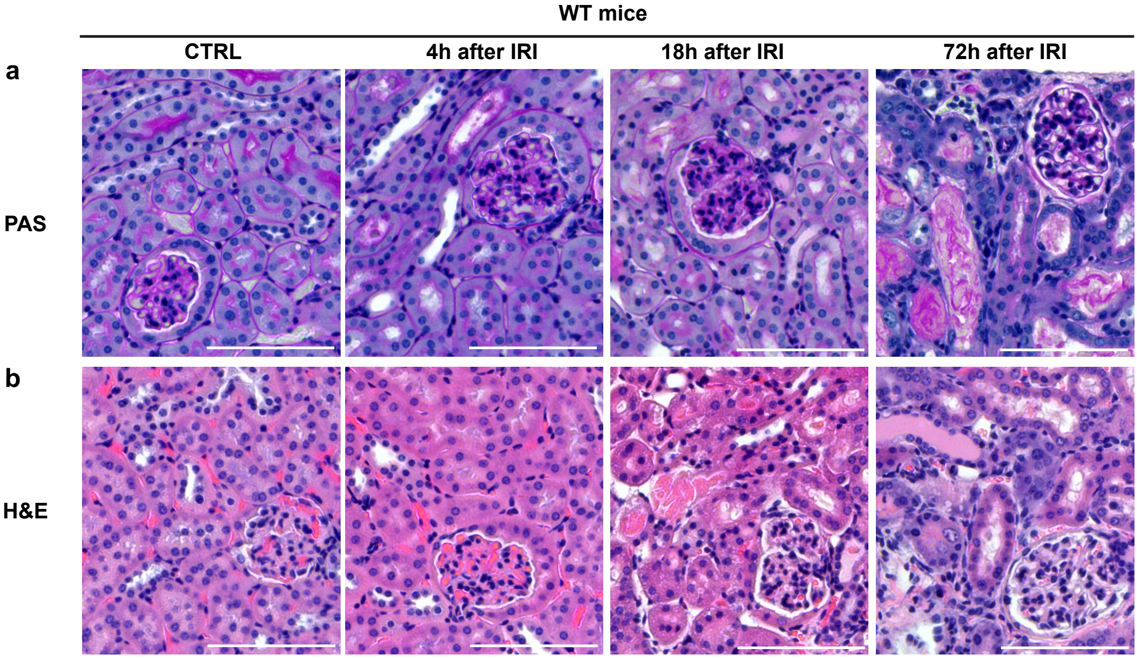

Supplementary Figure S2. Related to Figure 1. Histology of wild-type mice kidneys after ischemia/reperfusion injury (IRI).

(a, b) Periodic acid-Schiff (PAS) and Haemotoxylin and Eosin (H&E) staining revealed obvious morphological changes (inflammation, proteinuria, cell casts, tubule atrophy) 18 hours and 72 hours after IRI. n = 2-3. Scale bars = 100 µm.

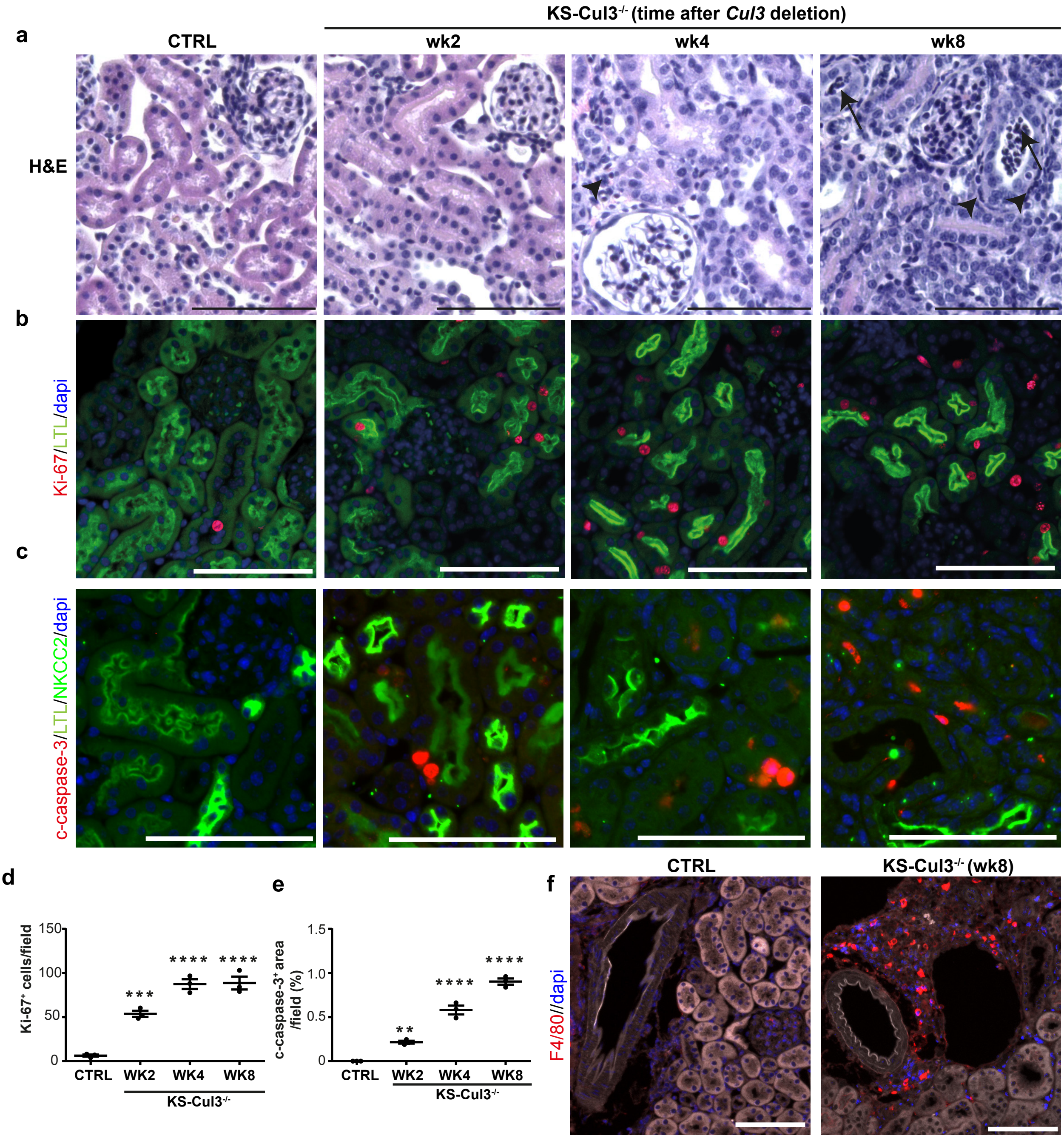

Supplementary Figure S3. Related to Figure 3 and 4. Tubule injury, proliferation and apoptosis upon *Cul3* deletion. (a) Haemotoxylin and Eosin (H&E) staining revealed cellular infiltration (arrowheads) and dilated tubules with intratubular debris (arrows) upon *Cul3* disruption. **(b)** Immunofluorescence revealed increased overall number of Ki-67$^+$ proliferating cells in kidney sections from KS-Cul3$^{-/-}$ mice. Ki-67$^+$ cells were predominantly in LTL$^+$ proximal tubules and interstitium. **(c)** Cleaved-caspase-3$^+$ apoptotic cells were mainly found in LTL$^+$ proximal tubules two weeks after *Cul3* deletion. As an example, no apoptotic cells were found in more distal segments such as Na^+-K^+-Cl^- cotransporter (NKCC2)$^+$ thick ascending limb. At later time points, apoptotic cells were also found in LTL$^-$ tubules. These tubules might represent injured proximal tubules without brush border and/or more distal tubules with apoptotic intratubular debris. **(d)** quantification of (b). **(e)** quantification of (c). **(f)** Perivascular infiltration of F4/80$^+$ macrophages upon *Cul3* deletion. n = 3. Scale bars = 100 µm. Mean values are shown ± SEM. In (d, e) asterisks show significant differences between control and each KS-Cul3$^{-/-}$ mice group. ***$P \leq 0.001$, ****$P \leq 0.0001$; Ordinary one-way ANOVA with Dunnett's multiple comparisons test.

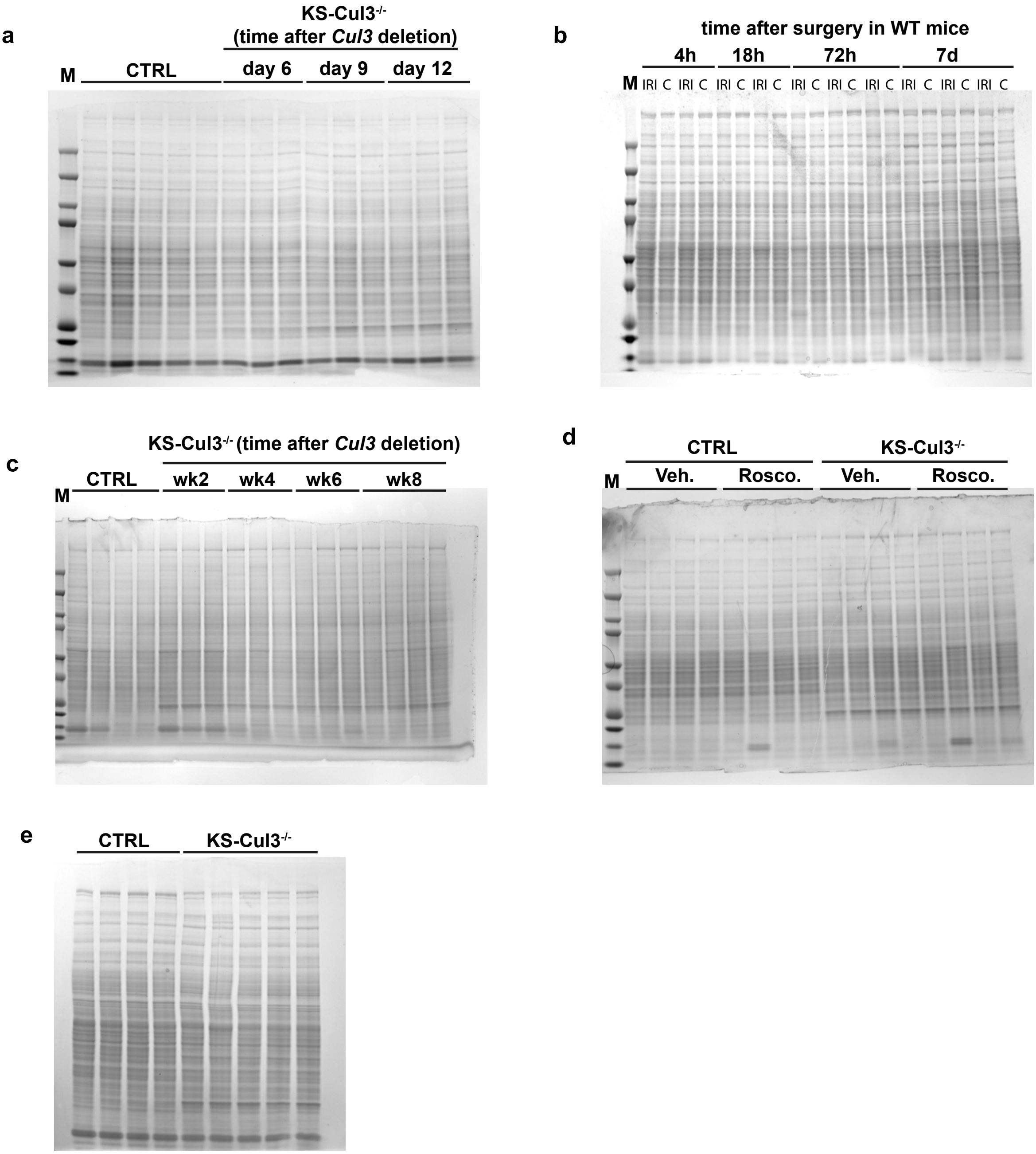

Supplementary Figure S4. Coomassie-stained gels related to immunoblots shown in main manuscript. (a) Coomassie gel related to Figs. 1a and 2a. Samples loaded on first and second lane were not used for immunoblots. **(b)** Coomassie gel related to Fig. 2g. **(c)** Coomassie gel related to Figs. 3a and 4a. Samples loaded on first and last lane as well as Cul3 knockout mice on week 6 were not used for immunoblots. **(d)** Coomassie gel related to Fig. 5b. **(e)** Coomassie gel related to Fig. 6h. Abbreviation: M, marker (Precision Plus Protein™ Kaleidoscope™ Prestained Protein Standards); CTRL, control; KS-Cul3$^{-/-}$, kidney-specific Cul3 knockout mice; WT, wild-type, wk, week; Veh., vehicle; Rosco., roscovitine

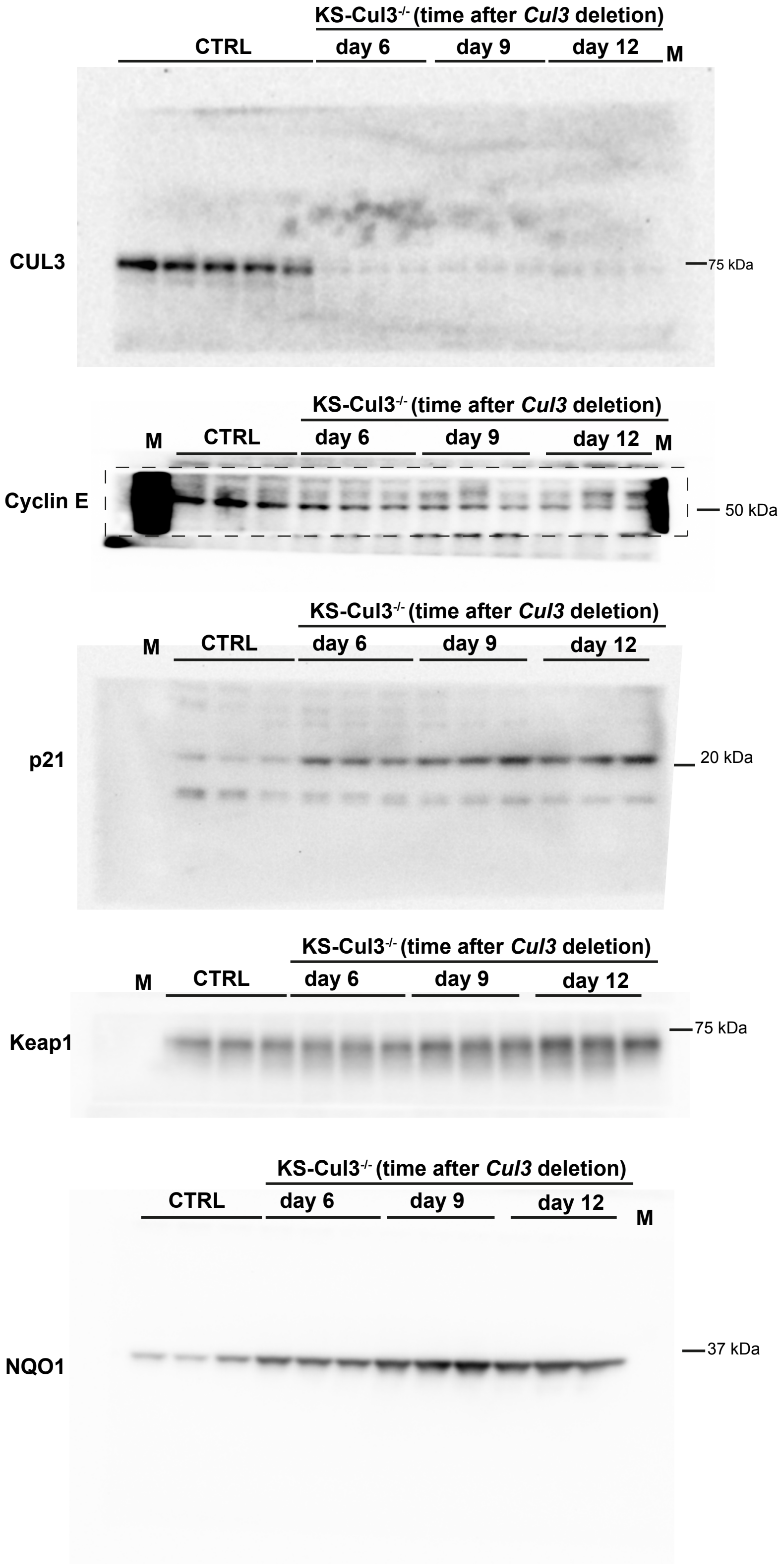

Supplementary Figure S5. Uncropped Western blot gels shown in main manuscript Fig. 1 and 2. M, marker (Precision Plus Protein™ Kaleidoscope™ Prestained Protein Standards).

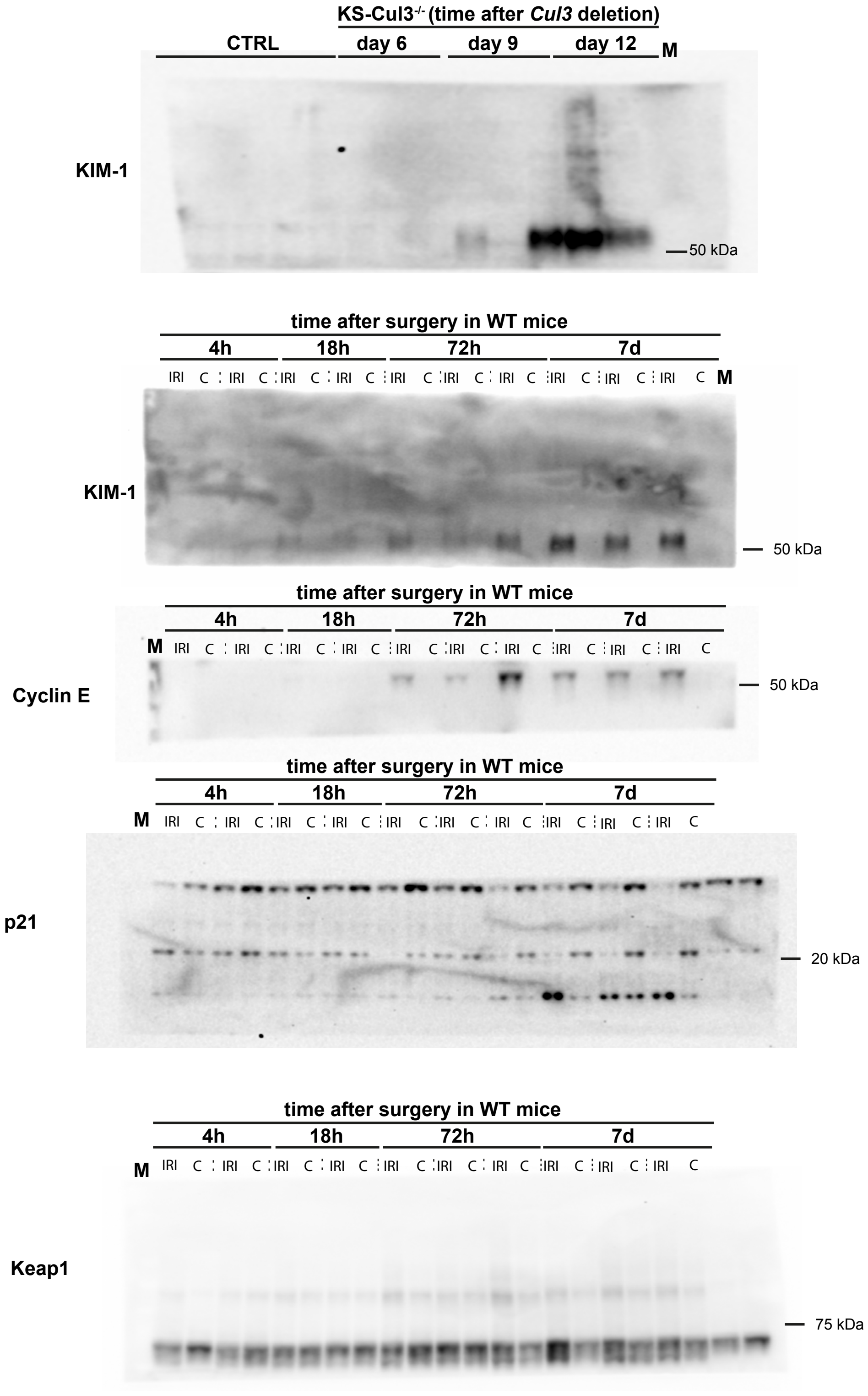

Supplementary Figure S6. Uncropped Western blot gels shown in main manuscript Fig. 2. M, marker (Precision Plus Protein™ Kaleidoscope™ Prestained Protein Standards); c or CTRL, control; IRI, ischemia-reperfusion injury; WT, wild-type.

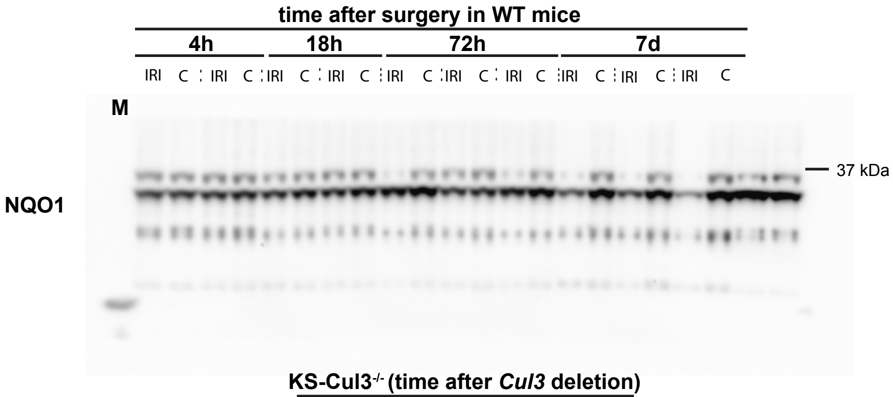

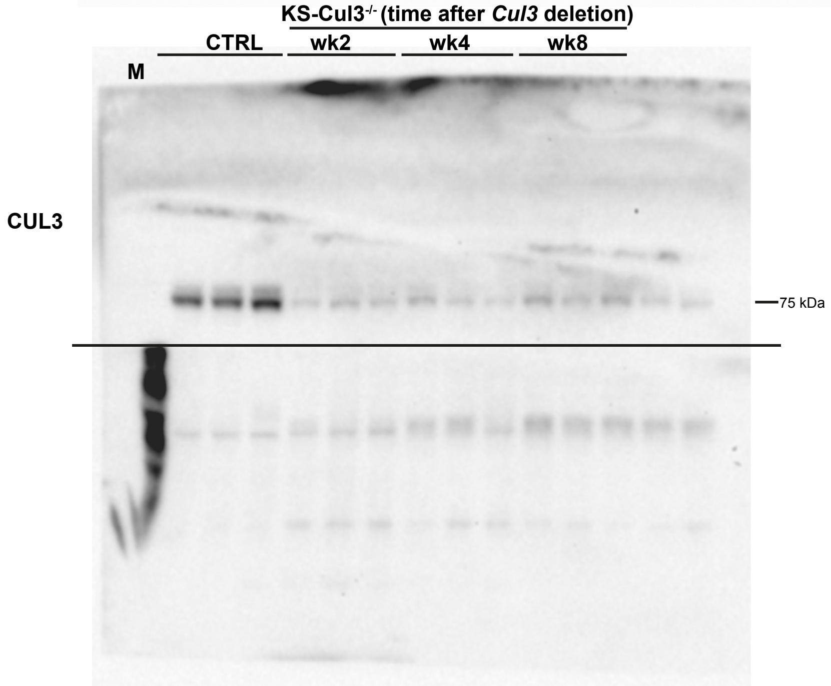

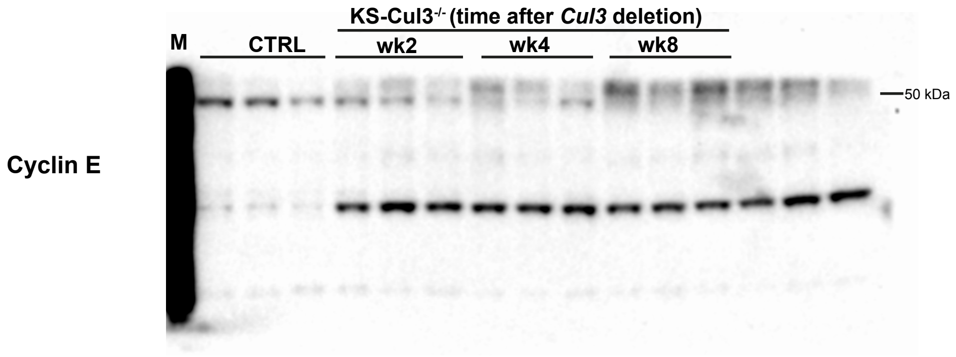

Supplementary Figure S7. Uncropped Western blot gels shown in main manuscript Fig. 2, Fig. 3 and Fig. 4. M, marker (Precision Plus Protein™ Kaleidoscope™ Prestained Protein Standards); c or CTRL, control; IRI, ischemia-reperfusion injury; WT, wild-type; wk, week.

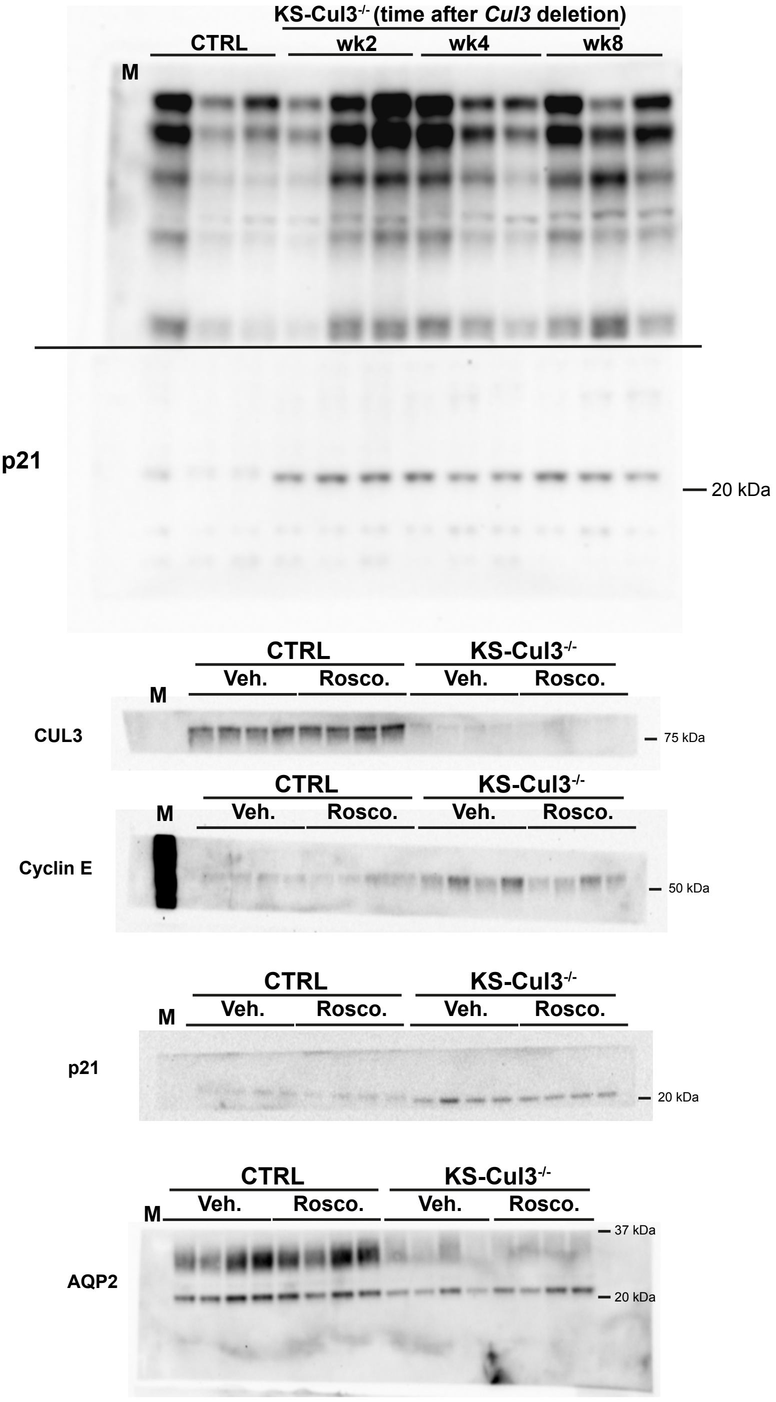

Supplementary Figure S8. Uncropped Western blot gels shown in main manuscript Fig. 4 and Fig. 5. M, marker (Precision Plus Protein™ Kaleidoscope™ Prestained Protein Standards); Veh., vehicle; Rosco., roscovitine; wk, week.

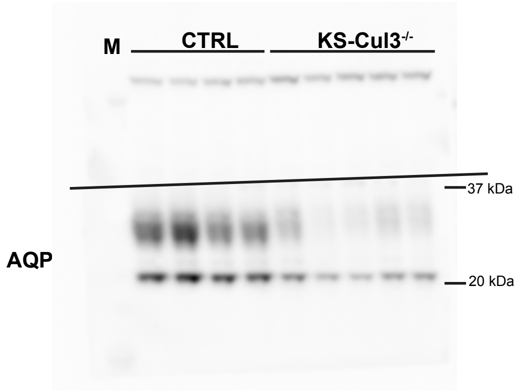

Supplementary Figure S9. Uncropped Western blot gels shown in main manuscript Fig. 6. M, marker (Precision Plus Protein™ Kaleidoscope™ Prestained Protein Standards); CTRL, control.

Supplementary Table S1. List of reagents and resources used in this study.

Reagent or Resource	Source, Catalog number	Dilution IF	Dilution WB
Antibodies			
Mouse anti-BrdU antibody	Roche, Cat#11296736001	1:120	
Rabbit anti-Cul3 antibody	Bethyl Laboratories, Cat#A301-109A		1:2000
Rabbit anti-Cul3 antibody	Sigma-Aldrich, Cat#C9745	1:120; 1:100 (IHC)	1:2000
Goat anti-KIM-1 antibody	R&D, Cat#AF1817	1:180	1:4000
Rabbit anti-NGAL antibody	Abcam, Cat#ab63929	1:200	
Mouse anti-F4/80 antibody	Santa Cruz Biotechnology, Cat#sc-377009	1:100	
Rabbit anti-CD3 antibody	Vector Laboratories, Cat#VP-RM01	1:500 (IHC)	
Rabbit anti-cleaved caspase-3	Cell signaling, Cat#9661S	1:120, 1:500 (IHC)	
Mouse anti-pH3 antibody	Santa Cruz Biotechnology, Cat#sc-374669	1:200	
Rabbit anti-Ki-67	Vector Laboratories, Cat#VP-RM04	1:120, 1:500 (IHC)	
Rabbit anti-Cyclin E antibody	Singer JD et al., 1999		1:1000
Rabbit anti-p21 (c19)	Santa Cruz Biotechnology, Cat#sc-397		1:1000
Anti-Actin, α-Smooth Muscle – Cy3	Sigma Aldrich, Cat#C6198	1:200	
Rabbit anti-Phospho-Histone H2A.X	Cell signaling, Cat#9718	1:100	
Rabbit anti-pT96/T101-NKCC2	Saritas T et al., 2013	1:4000	
Rat anti-Keap1	Millipore Sigma, Cat#MABS514		1:2000
Rabbit anti-NQO1	Abcam, Cat#ab34173		1:3000
Goat anti-PCNA	Sigma-Aldrich, Cat#SAB2502098	1:100	
Rabbit anti-PCNA	Abcam, Cat#ab2426	1:200	
Rat anti-CD44	BD Biosciences Pharmingen, Cat#BD553131	1:200	
Goat anti-Aquaporin 2	Santa Cruz Biotechnology, Cat#sc-9882	1:1000	1:4000
Rabbit anti-Collagen 1	Abcam, Cat#ab34710	1:200	
Rabbit anti-Fibronectin	Abcam, Cat#ab23750	1:200	
Chemicals, Peptides, and Recombinant Proteins			
Fluorescein labelled Lotus Tetragonolobus Lectin	Vectorlabs, Cat#FL-1321	1:200	
Coomassie G-250	Bio-Rad, Cat#161-0786		
FITC-Sinistrin	Fresenius-Kabi Austria		
Albuwell M	Exocell, Cat#1011		
Doxycycline	Alfa Aesar, Cat#J60579		
Antigen unmasking solution	Vector Laboratories, Cat#H-3300		
DAPI mounting medium	Thermo Fischer Scientific, Cat#P36971		
Paraformaldehyde	Thermo Fischer Scientific, Cat#O4042-500		
Roscovitine	Med Chem Express, Cat#HY-30237		
DMSO	Sigma, Cat#D4540		
Poly(ethylene glycol) 300	Sigma, Cat#202371		
Avidin/Biotin blocking kit	Vector Laboratories, Cat#SP-2001		
Peroxidase substrate kit	Vector Laboratories, Cat#SK-4100		
Vectastain ABC Kit	Vector Laboratories, Cat#PK-6100		

Hydrogen peroxide	Thermo Fischer Scientific, Cat#H323		
Methanol	Thermo Fischer Scientific, Cat#A412		
BSA	Sigma-Aldrich, Cat#A7906		
Sucrose	Thermo Fischer Scientific, Cat#15503022		
EDTA	Thermo Fischer Scientific, Cat#15576028		
EGTA	Millipore Sigma, Cat#41-005-0GM		
Sodium Orthovanadate	Millipore Sigma, Cat#508605		
Sodium fluoride	Millipore Sigma, Cat#106450		
Ditiothreitol	Millipore Sigma, Cat#20-265		
Phenylmethane sulfonyl fluoride	Millipore Sigma, Cat#7110-OP		
Aprotinin	Sigma-Aldrich, Cat#A6270		
Leupeptin	Thermo Fischer Scientific, Cat#78435		
Western Lightning Plus ECL	Perkin-Elmer, Cat#NEL103E001EA		
Experimental Models: Organisms/Strains			
Mouse: Pax8-rtTA/LC-Cul3$^{fl/fl}$	McCormick et al., 2014		
Mouse: Cul3^{flx}	The Jackson Laboratory, Stock No: 028349		
Mouse: C57BL/6J	The Jackson Laboratory, Stock No: 000664		
Software and Algorithms			
GraphPad Prism	https://www.graphpad.com/scientific-software/prism/		
Other			
Normal diet	Labdiet, Cat#5LOD		
